# Defining a Centiloid scale threshold predicting long-term progression to dementia in patients attending the memory clinic: an [^18^F] flutemetamol amyloid PET study

**DOI:** 10.1007/s00259-020-04942-4

**Published:** 2020-06-29

**Authors:** Bernard J. Hanseeuw, Vincent Malotaux, Laurence Dricot, Lisa Quenon, Yves Sznajer, Jiri Cerman, John L. Woodard, Christopher Buckley, Gill Farrar, Adrian Ivanoiu, Renaud Lhommel

**Affiliations:** 1 Institute of Neuroscience, Université Catholique de Louvain, Brussels, Belgium; 2 Neurology Department, Saint-Luc University Hospital, Av. Hippocrate, 10, 1200 Brussels, Belgium; 3 Gordon Center for Medical Imaging, Radiology Department, Massachusetts General Hospital, Harvard Medical School, Boston, MA USA; 4 Genetics Department, Saint-Luc University Hospital, Brussels, Belgium; 5 Department of Neurology, Charles University, 2nd Faculty of Medicine and Motol University Hospital, Prague, Czech Republic; 6 Department of Psychology, Wayne State University, Detroit, MI USA; 7 GE Healthcare, Amersham, UK; 8 Nuclear Medicine Department, Saint-Luc University Hospital, Brussels, Belgium; 9 Institute of Experimental and Clinical Research, Université Catholique de Louvain, Brussels, Belgium

**Keywords:** Amyloid PET, Mild cognitive impairment, AD dementia, Centiloids, Diagnostic accuracy

## Abstract

**Purpose:**

To evaluate cerebral amyloid-β(Aβ) pathology in older adults with cognitive complaints, visual assessment of PET images is approved as the routine method for image interpretation. In research studies however, Aβ-PET semi-quantitative measures are associated with greater risk of progression to dementia; but until recently, these measures lacked standardization. Therefore, the Centiloid scale, providing standardized Aβ-PET semi-quantitation, was recently validated. We aimed to determine the predictive values of visual assessments and Centiloids in non-demented patients, using long-term progression to dementia as our standard of truth.

**Methods:**

One hundred sixty non-demented participants (age, 54–86) were enrolled in a monocentric [^18^F] flutemetamol Aβ-PET study. Flutemetamol images were interpreted visually following the manufacturers recommendations. SUVr values were converted to the Centiloid scale using the GAAIN guidelines. Ninety-eight persons were followed until dementia diagnosis or were clinically stable for a median of 6 years (min = 4.0; max = 8.0). Twenty-five patients with short follow-up (median = 2.0 years; min = 0.8; max = 3.9) and 37 patients with no follow-up were excluded. We computed ROC curves predicting subsequent dementia using baseline PET data and calculated negative (NPV) and positive (PPV) predictive values.

**Results:**

In the 98 participants with long follow-up, Centiloid = 26 provided the highest overall predictive value = 87% (NPV = 85%, PPV = 88%). Visual assessment corresponded to Centiloid = 40, which predicted dementia with an overall predictive value = 86% (NPV = 81%, PPV = 92%). Inclusion of the 25 patients who only had a 2-year follow-up decreased the PPV = 67% (NPV = 88%), reflecting the many positive cases that did not progress to dementia after short follow-ups.

**Conclusion:**

A Centiloid threshold = 26 optimally predicts progression to dementia 6 years after PET. Visual assessment provides similar predictive value, with higher specificity and lower sensitivity.

**Trial registration:**

Eudra-CT number: 2011-001756-12

## Introduction

Positron emission tomography (PET) using radiotracers specific for amyloid-β(Aβ) plaques has been a breakthrough for Alzheimer’s disease (AD) research [1]. Several PET studies have demonstrated that Aβ plaques are detectable in the brain of non-demented older adults [2]; and that high Aβ-PET signal is associated with subsequent cognitive decline [3–5] and greater risk for progression to dementia [6, 7]. The results of these research studies make Aβ-PET a promising biomarker for Memory Clinics to evaluate the risk of individual patients to develop dementia. So far, three Aβ tracers (florbetapir, florbetaben, and flutemetamol) have been approved for clinical use. Specifically, [^18^F] flutemetamol is approved in the USA and the European Union to visually rule out Aβ as the probable cause of the cognitive deficits. PET semi-quantitation could potentially increase sensitivity and detect Aβ earlier than visual readouts [8]; but until recently, the use of multiple radiotracers and the lack of standardization in semi-quantitative measurements have hampered the field by limiting between-study comparability. Consequently, PET quantitation is commonly used in research but is not routine in clinical practice.

The Centiloid project was designed to address this issue by proposing a standardized Aβ-PET processing pipeline and a method to transform resulting PET signal measures obtained with different radiotracers into a common unit, called “Centiloid” [9]. The Centiloid Aβ-PET scale is anchored at 0 and 100 Centiloids, with 0 Centiloid score reflecting a definitively Aβ-negative brain (originally calculated as the average value of a group of healthy subjects below the age of 45) and 100 Centiloids reflecting the average signal observed in patients with typical mild or moderate AD dementia. This harmonized method (originally anchored to [^11^C] PiB but now applicable to [^18^F] tracers after calibration) has great potential to produce cohesive and comparable results from disparate clinics across the world.

The present study compared visual and semi-quantitative assessments of [^18^F] flutemetamol PET data in a cohort of normal older adults and non-demented patients recruited at a single Memory Clinic. We used the overall predictive value to assess progression to dementia after a long-term follow-up as our standard of truth and tested different Centiloid thresholds. As a secondary objective, we evaluated whether PET semi-quantitation could help detecting visually negative cases that would subsequently progress to visually positive in a subset of participants with longitudinal PET data.

## Methods

### Participants—see Table 1 for demographic and cognitive data

One hundred sixty-one older adults (age, 54–86) were screened and 160 were included in the study. One patient was excluded from all analyses because of a PS1 (G384A) mutation. Thirty-one participants were clinically normal (CN) controls, recruited by advertisement, and 129 were non-demented patients who attended our clinic between January 2012 and December 2018. Inclusion criteria were cognitive complaints, confirmed by a relative and a neurologist, and a mini-mental state exam (MMSE) score ≥ 24/30. Exclusion criteria were dementia (DSM-IV criteria), focal brain lesions, major depression or psychiatric diseases, alcohol or drug abuse, and autosomal dominant mutations (PS1, PS2, and APP), which were systematically searched for in patients younger than 65. Mild cognitive impairment (MCI) was defined as a performance below the 10th percentile (composite *z*-score < −1.3) of an independent age-matched control group in any of the following cognitive domains [10]: memory (free and cued selective reminding test), language (Lexis naming test, the 2-min letter fluency for “P,” and the 2-min category fluency for animals), executive functions (trail making test and Luria’s graphic sequences), and visuospatial functions (clock drawing test and the praxis part of the CERAD battery). Patients performing above the 10th percentile in all cognitive domains were classified as subjective cognitive decliners (SCD). Participants had annual clinical follow-up including MMSE and performed the neuropsychological evaluation every other year after baseline. Two neurologists (B.H./A.I.) with more than a 10-year experience of Memory Clinics made dementia diagnoses using all the clinical and cognitive data available.

**Table 1:** Characteristics of the participants

	All	Visually negative	Visually borderline	Visually positive
Number included	160	91	7	62
Age (years)	71.4 ± 7.5 (54–86)	70.6 ± 7.2 (54–86)	71.0 ± 8.8 (59–82)	72.4 ± 7.9 (54–83)
ε4 carriers: number (%)	66 ε4 (46%) 16 missing	19 ε4 (23%) 8 missing	4 ε4* (67%) 1 missing	43 ε4** (78%) 7 missing
Education (years)	14.4 ± 4.6 (6–20)	14.0 ± 4.7 (6–20)	16.3 ± 2.9 (12–18)	14.7 ± 4.5 (6–18)
Female: number (%)	81 ♀ (51%)	46 ♀ (51%)	4 ♀ (57%)	31 ♀ (50%)
Baseline MMSE score (/30)	27.3 ± 1.8 (24–30)	27.9 ± 1.7 (24–30)	27.7 ± 1.4 (26–30)	26.4 ± 1.6** (24–30)
Clinical diagnoses (CN/SCD/MCI)	31/35/94	26/25/40	3/2/2	2/8/52**
Neocortical flutemetamol SUVr	1.50 ± 0.33 (0.91–2.44)	1.25 ± 0.09 (0.91–1.47)	1.48 ± 0.11** (1.28–1.62)	1.87 ± 0.21** (1.43–2.44)
Centiloids	36.2 ± 41.2 (− 33–140)	3.9 ± 11.7 (−33–44)	39.9 ± 8.5** (29–53)	82.4 ± 41.2** (45–140)
Number with long clinical follow-up^$^	98	58	2	38
Clinical diagnoses (CN/SCD/MCI)	28/24/46	24/19/15	2/0/0	2/5/31 **
Centiloids	36.1 ± 41.2 (− 32–140)	5.4 ± 12.3 (− 32–44)	31.1 ± 3.3** (29–33)	83.2 ± 21.6** (49–140)
Number of patients demented after follow-up (%)	46 (47%)	10 (17%)	1 (50%)	35 (92%)
Clinical follow-up duration (years) ^$^	4.8 ± 1.9 (1.1–8.0)	5.8 ± 1.6 (1.1–8.0)	5.7 ± 2.1 (4.2–7.2)	3.2 ± 1.3 ** (2.0–6.4)
Number followed using PET	34	33	1	0
PET follow-up duration (years)	3.1 ± 0.9 (1.5–6.2)	3.1 ± 0.9 (1.5–6.2)	2.8	/
Number of patients visually positive after follow-up (%)	4 (12%)	3 (9%)	1 (100%)	/

Mean ± SD (min-max), *p < 0.05, **p < 0.001 compared to the visually negative group. One patient was recruited but excluded from the study because of a presenilin 1 mutation

^$^Clinical follow-up duration is at least 4 years for the patients who did not progress to dementia

### [^18^F] Flutemetamol PET imaging

At study start, [^18^F] flutemetamol (now marketed as ™Vizamyl, GE Healthcare) was proposed as an investigational medicinal radiopharmaceutical drug, being studied clinically as an Aβ-imaging agent. Ninety minutes after intravenous injection of [^18^F] flutemetamol (target activity 185 ± 5 MBq), a 30-min list-mode PET/CT acquisition was performed on a Philips Gemini TF (Philips Healthcare) and reconstructed as a dynamic scan of 6 × 5 min with 2-mm isometric voxels including attenuation, scatter, decay corrections, and time-of-flight information using the manufacturer’s standard reconstruction algorithm. No partial volume correction was applied to the data.

To represent routine clinical conditions as closely as possible, visual qualitative assessments were only performed by one qualified Nuclear Medicine physician (RL), following criteria defined in a dedicated training program provided by GE Healthcare.

PET semi-quantitative data were computed both using an in-house SUVr pipeline using neocortex uptake scaled to cerebellar gray [10–13] and using a dedicated Centiloid pipeline. Standardized uptake values (SUV_Centiloid_) were computed using the PNEURO software (v3.9) (PMOD LLC Technologies, Switzerland) on the averaged dynamic scan, after visual check of the dynamic sequence and motion correction if required. The PMOD Centiloid atlas (cortex and whole cerebellum volumes-of-interest (VOI), applied on the MNI ICBM152T1 template) was used after MRI segmentation, PET/MRI co-registration, and transposition of the PET/MRI datasets into the stereotaxic MNI reference space (maximum probability atlas workflow). 3D-T1 MRI was acquired for each participant on a 3 T MRI (Philips Achieva). SUVr was defined as the SUV ratio of the Centiloid cortical VOI and either cerebellar gray (SUVr_in-house_) or whole cerebellum (SUVr_Centiloid_).

### Transposition of [^18^F] flutemetamol SUVr_Centiloid_ to Centiloid scale value

We followed the sequential calibration steps to convert [^18^F] flutemetamol SUVr _in-house_ to the Centiloid scale, initially proposed for [^11^C] PiB [1], then endorsed by the Global Alzheimer’s Association Interactive Network (GAAIN, http://www.gaain.org/centiloid-project) to help centers calibrate and report their data regardless the tracer and semi-quantitation method used. The results of these different iterative calibration steps using PMOD NEURO 3.9 matched the calibration program requirements and have been previously reported in this [14, 15] and other [16] samples. The direct conversion of SUVr_Centiloid_ to Centiloid values was Centiloid = 116.0 × SUVr_Centiloid_ − 113.9. As both measures were equivalent (*R*^2^ = 1.0), we only reported Centiloid values in the results section, and no SUVr_Centiloid_. Consequently, we refer to the SUVr_in-house_ as SUVr in the “Results” section.

### Statistics

We compared visually positive and negative cases using *t* tests and chi-square, and we computed receiver operating characteristic (ROC) curves to identify the most accurate PET threshold to predict subsequent progression to dementia. In this analysis, we initially focused on participants with at least 4 years of follow-up data available to ensure the long-term validity of predictive values. We computed statistics in Matlab R2017b and reported two-tailed *p* values.

## Results

### Qualitative and semi-quantitative Centiloid assessments

**Fig. 1:**
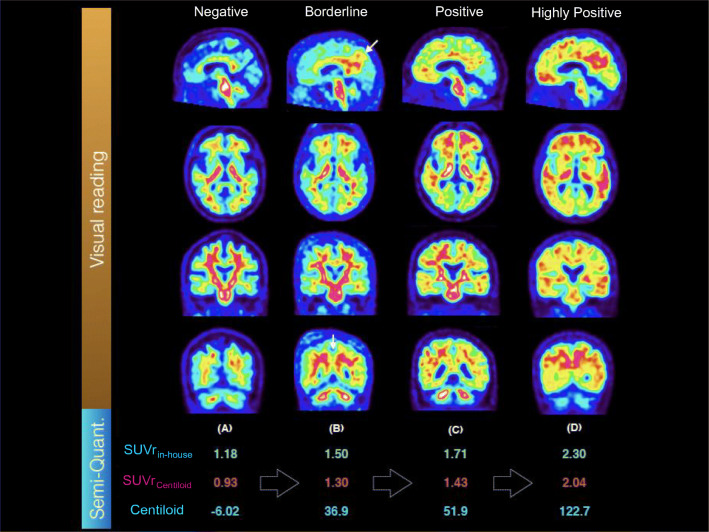
Illustrative PET images with visual (top) and semi-quantitative assessments (bottom). Four cases have been selected illustrating **a** a negative, **b** a borderline, **c** a positive, and **d** a highly positive PET reading. Visually borderline cases are overall negative with positivity visible in the posterior cingulate region. Because PET results were disclosed to patients, we did not feel confident to classify these scans as positive before having follow-up. Positive and highly positive cases were treated similarly

Qualitative assessment of the PET images categorized 26 CN (of 31, 84%), 25 SCD (of 35, 71%), and 40 MCI patients (of 94, 43%) as visually negative. Another three CN (10%), two SCD (6%), and two MCI patients (2%) were visually borderline, i.e., the image was visually negative in frontal, parietal, and temporal neocortex, and striatum; however, a thick subcortical uptake raised doubts about possible cortical positivity and/or posterior cingulate tags were observed (Fig. 1). The remaining two CN (6%), eight SCD (23%), and 52 MCI patients (55%) were visually positive without any doubts. Demographics were similar across groups, but visually positive cases performed worse on the MMSE than visually negative cases. They were also more likely to be ε4 carriers (Table 1).

**Fig. 2:**
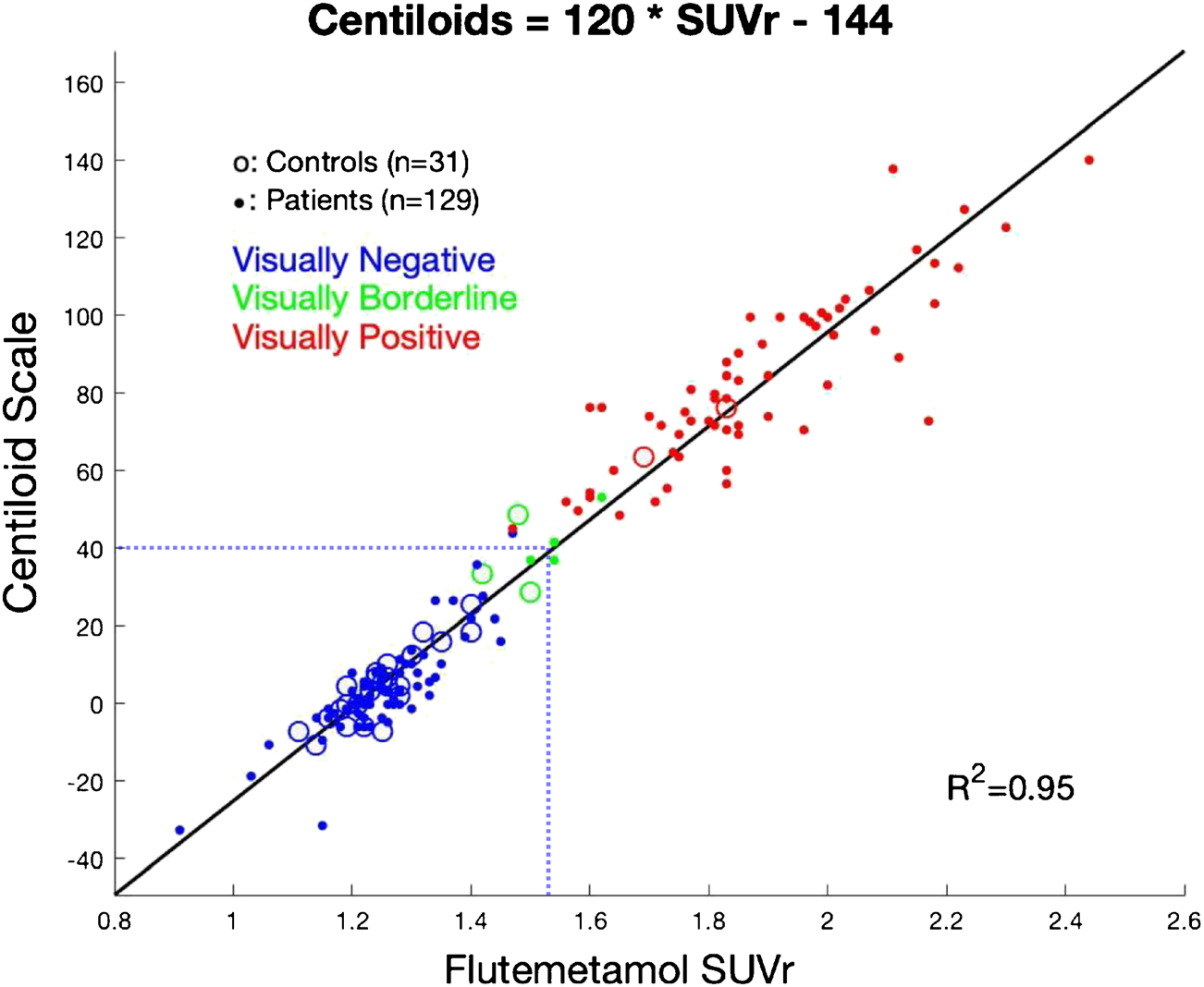
Scatterplot demonstrating the linear relationship between PET data expressed using our in-house [^18^F]-flutemetamol SUVr and the same PET data expressed in the Centiloid scale. Visual threshold corresponded to Centiloid = 40 (SUVr = 1.53), indicated by the blue dotted line. The direct conversion of SUVr_Centiloid_ to Centiloid values was Centiloid = 116.0 × SUVr_Centiloid_ − 113.9. The conversion of our in-house SUVr (using cerebellar gray as the reference region) to Centiloid values was: Centiloid = 120.2 × SUVr_in-house_ − 144.5

Transforming our in-house [^18^F] flutemetamol SUVr into the Centiloid scale resulted by data fitting in the following equation: Centiloid = (120.2 × SUVr _in-house_) – 144.5, with a strong correlation between both measures (Fig. 2, *R*^2^ = 0.95). After excluding borderline cases, qualitative and quantitative assessments matched in all but one case (> 99%), when setting the threshold between Centiloid = 37 and Centiloid = 43 [12, 17]. No participants with clearly negative or positive reads felt in that range, we thus arbitrarily set the visual threshold at Centiloid = 40. The visually borderline cases had close-to-threshold quantitative assessments (Fig. 2, green circles), four were below the Centiloid = 40 threshold and three were above-threshold, making the overall visual-quantitative agreement = 97% if borderline cases were considered as negative, and = 98% if they were considered as positive.

### Progression to dementia

Clinical outcome after at least 9 months of follow-up was available for 123 study participants. Twenty-five study participants only had short follow-up (< 4 years, median = 2 years) and did not progress to dementia during this period: We initially excluded them, because we could not ascertain whether they would become demented later on. Of the remaining 98 participants, 52 were non-demented after a median 6-year follow-up duration (min, 4.0 – max, 8.0). Forty-six participants, including two CN (of 28, 7%), seven SCD (of 24, 29%), and 37 MCI (of 46, 80%) patients progressed to dementia, after a median follow-up time of 2.5 years (min, 1.1 – max, 6.4, Table 1).

**Fig. 3:**
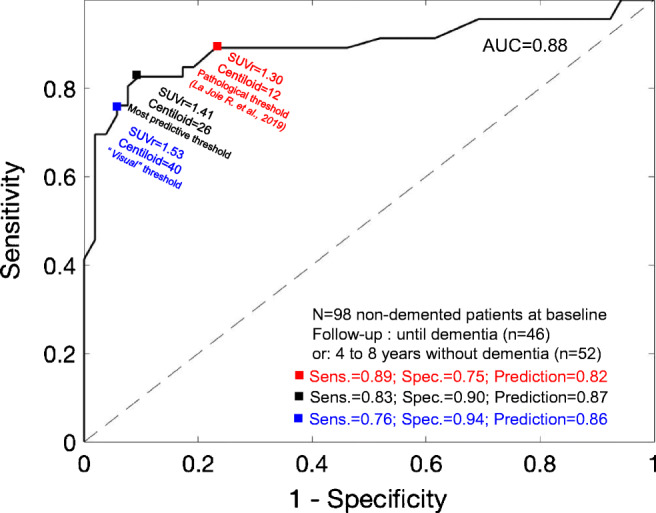
Receiver operating characteristic (ROC) curves using baseline [^18^F]-flutemetamol PET data to predict dementia progression after a median six-year follow-up. The most predictive threshold was observed at Centiloid = 26 (black square). Visual threshold (blue square) and a threshold based on pathological literature (red square) are also provided

**Fig. 4:**
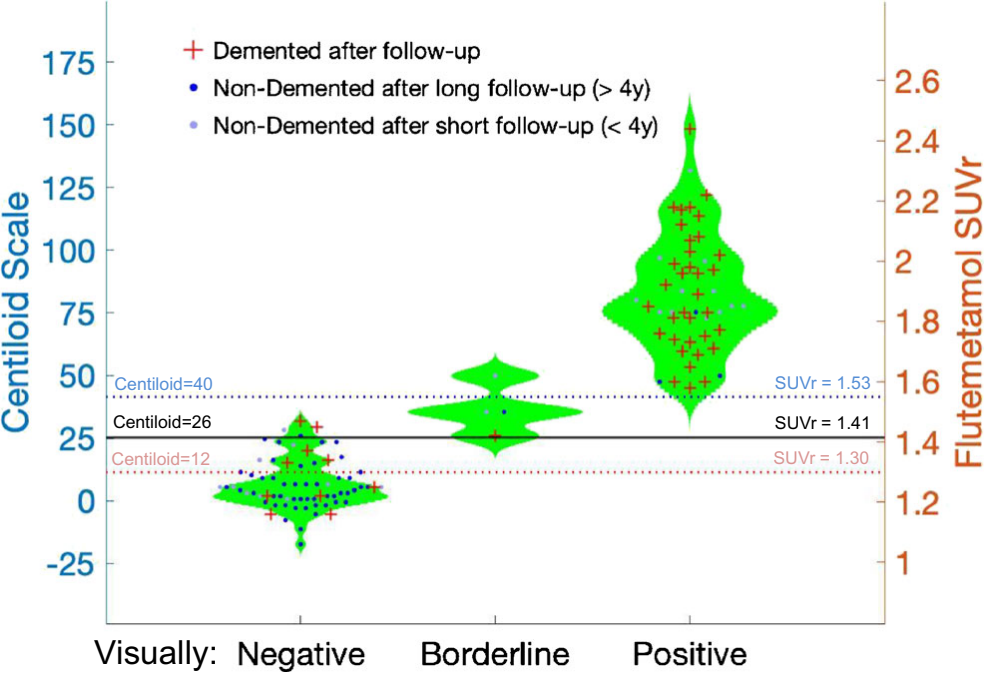
Violin plots presenting PET quantitative data according to visual readings, clinical outcomes after follow-up, and follow-up duration

We first observed that the baseline Centiloid values predicted dementia progression in a logistic regression adjusted for age, sex, ε4 carriage, and MMSE at baseline (*p* = 0.001). We then computed a ROC curve predicting dementia using baseline [^18^F] flutemetamol data and observed an area under the curve = 0.88 (Fig. 3, no covariate). The threshold providing the highest overall predictive value (87%) was observed at Centiloid = 26 (area under the ROC curve: AUC = 88%). Using this threshold provided a sensitivity of 83% (CI 95, 69–92%), a specificity of 90% (79–97%), a positive predictive value (PPV) of 88% (77–95%), and a negative predictive value (NPV) of 85% (76–92%), i.e., only 8 of 55 participants (15%) with below-threshold PET uptake progressed to dementia, while 38 of 43 participants (88%) with above-threshold PET uptake progressed to dementia 6 years after PET (Fig. 4). In comparison with a Centiloid = 26 quantitative threshold, visual assessment (which was equivalent to using a Centiloid threshold = 40) provided a higher PPV = 92% (79–97%) and a lower NPV = 81% (73–88%) (overall prediction = 86%).

When separately analyzing the MCI and CN/SCD (that were grouped together due to the small numbers of progressors), using a threshold set at Centiloid = 26, we observed a higher PPV in Aβ + MCI (29 of 31 progressed, PPV = 94% (79–99%)) than in Aβ + CN/SCD (8 of 13 progressed to dementia, PPV = 62% (32–86%)). In contrast, in Aβ-negative individuals, the NPV was higher in CN/SCD (1 SCD of 39 CN/SCD progressed to dementia, NPV = 97% (87–99%)) than in MCI (8 of 15 progressed, NPV = 47% (19–73%)). The overall predictive value of Aβ-PET was marginally higher in CN/SCD (89%, (77–96%)) than in MCI (78%, (64–89%)), due to the high proportion of Aβ-MCI who progressed to an amnestic “AD-like” dementia.

We then tested the Centiloid threshold derived from 179 autopsy-confirmed [^11^C] PiB PET cases [18] (Centiloid = 12), which decreased the PPV = 75% (66–84%) without increasing much the NPV = 88% (77–95%), providing a lower prediction = 82%.

**Fig. 5:**
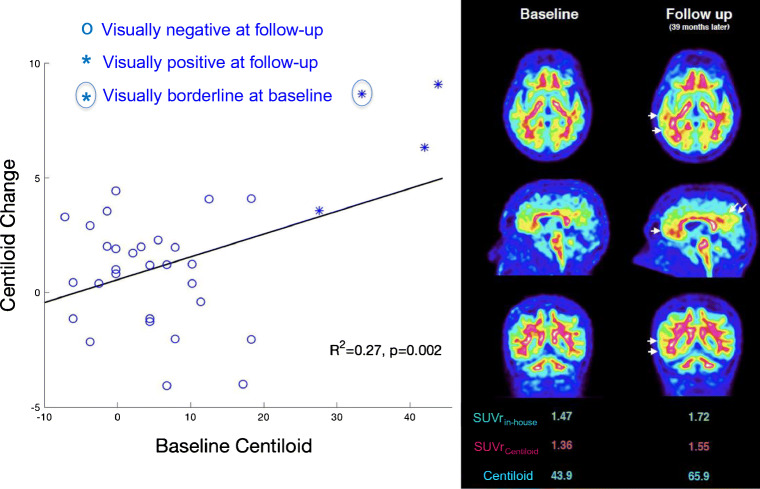
Baseline Aβ-PET predicts subsequent change in PET data, driven by individuals with positive quantitation at baseline. Left: fast accumulation (> 5 Centiloids per year) was only observed in participants with Centiloid ≥ 26 at baseline. Note that no patients had a baseline Centiloid between 20 and 25. All participants but one (borderline) had a visually negative baseline PET, although four participants (designated with ×) had positive quantitation (Centiloid between 27.6 and 43.9). Right: illustrative PET images of a participant who demonstrated increased Aβ-PET signal during follow-up

When including the 25 participants with only limited follow-up (< 4 years), we also observed a best threshold at Centiloid = 26, but the PPV and specificity of [^18^F] flutemetamol were decreased (PPV = 67% (58–76%); NPV = 88% (80–93%); sensitivity = 83% (69–92%); specificity = 76% (66–86%); AUC = 79%), reflecting the many positive cases who did not progress to dementia after shorter follow-ups (Fig. 5, gray dots). Because it provided the best overall predictive value, we subsequently used Centiloid = 26 as threshold for PET positivity.

### Progression to Aβ positivity

Thirty-four participants with a visually negative (or borderline, *n* = 1) baseline PET had a second [^18^F] flutemetamol PET after a median 3-year follow-up (min, 1.5 – max, 6.2). Four participants had a positive quantification at baseline (27 ≤ Centiloid ≤ 42) although visual assessment was negative (*n* = 3) or borderline (*n* = 1). At follow-up, the four PET images were visually positive, whereas no participants with negative quantification at baseline had a positive scan at follow-up. The average change was different from zero in the group with baseline positive quantification (CI 95, + 3 to + 11 Centiloids/year, *p* = 0.01), but it was not in the group with negative quantification (CI 95, − 2 to + 2 Centiloids/year, *p* = 0.93). We observed greater change in the participants with positive quantification compared to negative quantification, after adjusting for age, sex, ε4 carriage, and MMSE at baseline (*p* = 0.01). Excluding the participant with a borderline visual assessment at baseline did not modify the result (*p* = 0.05), demonstrating that Aβ-PET quantification could facilitate detection of persons with visually negative PET scans who will progress to visually positive scans in the next 3 years. Finally, we observed that the association between baseline Centiloid and subsequent change in Centiloid (Fig. 5, *p* = 0.002) was driven entirely by the four participants with baseline Centiloid > 26 who progressed from a negative (or borderline) scan at baseline to a visually positive scan at follow-up.

## Discussion

In this study, we sought to compare the long-term predictive values of qualitative, visual assessments and quantitative, Centiloid assessments of [^18^F] flutemetamol Aβ-PET images acquired in a monocentric cohort of non-demented participants attending the Memory Clinic. By assessing true and false positive in amyloid PET-diagnosed patients, this prospective diagnostic accuracy study accomplishes the primary aim of the 4th phase of an AD biomarker development [19, 20]. We observed that a threshold set at Centiloid = 26 best discriminated participants who will progress to dementia from participants who will remain clinically stable 6 years after PET. Using a Centiloid = 26 threshold increased the predictive value of clinical diagnoses: Overall, 80% of the MCI and 17% of the CN/SCD progressed to dementia after 6 years. Among the Aβ + (PPV), 94% of the MCI and 62% of the CN/SCD progressed, while among the Aβ-negative (NPV), 47% of the MCI and 97% of the CN/SCD remained clinically stable.

Visual assessment provided a similar, yet marginally lower, overall predictive value compared to a quantitative threshold set at Centiloid = 26. Using positive visual assessments as Aβ-PET readouts resulted in higher specificity (94% vs. 90%) and lower sensitivity (76% vs. 83%) for subsequent dementia compared to Centiloid assessments. Of note, only eight participants (5%) had a baseline Centiloid ≥ 26 but were not classified as visually positive. Among these cases, three progressed to dementia, two remained stable for longer than 6 years, and three were only followed for 2 years and were not included for deriving predictive values (Fig. 4). Therefore, the overall predictions of quantitative (87%) and visual (86%) assessments only differed by one of the 98 participants (+ 1%, not significant). A previous study with post-mortem validation in 78 cases did not observe either different accuracy between visual and quantitative Aβ-PET assessments [21]. Larger studies are needed to further evaluate the added value of quantitative over visual assessments, particularly in discordant cases, or to increase the sensitivity of early Aβ detection. We are only aware of one large study that specifically looked into the predictive power of visual versus quantitative Aβ-PET measurements: In 401 MCI participants from the ADNI cohort [22], the authors reported that visual reads had higher specificity (96% vs. 90%) and lower sensitivity (79% vs. 85%) than the SUVr values, with similar overall predictive power. However, follow-up length was relatively short (1.6 years), preventing a valid comparison of the utility of quantitation and visual reads for predicting how discordant cases evolve over a longer follow-up period. In a longer study (up to 3 years) [5], flutemetamol PET images were found positive in 52 of 81 amnestic MCI who converted to AD dementia (sensitivity, 64%), while they were negative in 99 of 143 non-converters (specificity, 69%); however, this study did not compare quantitation and visual assessments.

Importantly, we observed that the predictive value of a positive scan significantly dropped from 88 to 67% when including the participants who were followed for short periods (< 4 years), highlighting the importance of follow-up duration for establishing a clinical standard-of-truth. Of note, the positive predictive value is excluded from the routine approved label for Aβ-PET imaging (https://www.ema.europa.eu/en/documents/product-information/vizamyl-epar-product-information_en.pdf) since studies used for registration purposes were deemed to have too short a follow-up period for this claim to be made. Studies therefore as the one described in this paper with a longer term follow-up could contribute to the body of data highlighting the risk of clinical progression in the presence of Aβ pathology, that is, according to our data, 67% risk of progressing to dementia after 4 years, and 88% after 6 years.

We also observed lower PPV when using a Centiloid = 12 threshold, derived from Aβ assessment at post-mortem [18] (moderate to frequent CERAD plaque count). It is possible that participants with early Aβ, as indicated by a Centiloid in the 12–25 range, will progress to dementia many years after PET and that our clinical endpoint did not capture the slowly developing preclinical pathological process. Therefore, longer studies, up to a decade or more, are needed to accurately evaluate the impact of early Aβ burden on subsequent progression to dementia [23, 24]. Nevertheless, our longitudinal PET study did not observe any change in participants with sub-threshold signal, nor did the larger Mayo Clinic Study of Aging (Centiloid threshold = 19) [25], suggesting that an early Aβ-PET signal close to Centiloid = 12 is hardly distinguishable from noise. Interestingly, in this same PET autopsy study [18] as well as in a previous report [26], a higher threshold (Centiloid = 24) was observed against intermediate to high levels of AD neuropathological changes including thus both Aβ and tau lesions as a gold standard (unlike CERAD plaque count), which may be closer to a dementia endpoint. A recent paper observed that a Centiloid threshold = 21 detected moderate or frequent plaque density while Centiloid = 10 was optimal for excluding neuritic plaques [27]. They also observed higher threshold (Centiloid = 49) against intermediate to high levels of AD neuropathological changes. Consistently in CSF studies, PET thresholds obtained against CSF Aβ_42_ are low, at Centiloid = 12, while PET thresholds obtained against CSF total tau, phosphorylated tau, or tau/Aβ_42_ ratio are in the range of 25–33 [28]. Several studies using various endpoints (subsequent dementia, change in PET signal, CSF, histopathology [29]) generally converge around a Centiloid threshold = 26 (± 7), which appears to discriminate most accurately between those individuals on an AD trajectory from those who are not. Lower cut-off values can be used to detect incipient Aβ pathology, but these are not associated with progression to AD dementia, even after a median follow-up of 6 years.

From a clinical imaging standpoint, previous studies using visual ratings with [^11^C] PiB [30], [^18^F] flutemetamol [31], [^18^F] florbetaben [21], and [^18^F] florbetapir [32] showed good inter-rater reliability, especially among experienced readers. With the addition of quantitative information to visual reads, most raters change from “non-elevated” visual reads to “elevated” reads [8]. For experienced readers, the improvement in accuracy and confidence is likely restricted to a few borderline, but clinically relevant, cases [32] indicating that for the majority of images there is high concordance between visual interpretation and quantitative analysis. We only have one experienced nuclear physician at our center to perform visual reads, preventing us from evaluating inter-rater reliability. Still, we can confirm that using Centiloid = 26 as a threshold reclassified all our visually borderline cases (100%) and five of 91 (5.5%) negative visual reads to positive, while no visually positive cases were reclassified. Using Centiloid = 26 as a threshold also allowed detection of visually negative cases who progressed to visually positive at a subsequent scan, on average 3 years later. We therefore see the potential for the use of semi-quantitative assessment, either SUVr or a standardized metric such as the Centiloid scale, for increasing the sensitivity and the reliability over time of Aβ-PET interpretation in non-demented patients attending Memory Clinics. Future work will further investigate how regional PET signal—assessed visually or using quantitation—could contribute to dementia prediction [6, 33].

In conclusion, our results indicate that augmentation of visual interpretation of [^18^F]-flutemetamol Aβ-PET images with semi-quantitative, SUVr, or Centiloid information improves the sensitivity of visual assessment in some negative, and visually borderline, cases. While SUVr may be more readily available, the Centiloid scale offers easier comparison with other centers. When semi-quantitation is not available, we recommend considering visually borderline cases as positive. As visual and quantitative assessments provide the same classification for most patients, larger studies would be needed to demonstrate statistical differences between those assessments.
